# Prevalence and associated risk factors of malaria among febrile under-five children visiting health facilities in Ziquala district, Northeast Ethiopia: A multicenter cross-sectional study

**DOI:** 10.1371/journal.pone.0276899

**Published:** 2022-10-27

**Authors:** Habtu Debash, Habtye Bisetegn, Hussen Ebrahim, Daniel Getacher Feleke, Alemu Gedefie, Mihret Tilahun, Agumas Shibabaw, Endris Ebrahim, Mesfin Fiseha, Getu Abeje

**Affiliations:** 1 Department of Medical Laboratory Science, College of Medicine and Health Sciences, Wollo University, Dessie, Ethiopia; 2 Department of Microbiology, Immunology and Parasitology, College of Medicine and Health Sciences, Addis Ababa University, Addis Ababa, Ethiopia; 3 Department of Medical Laboratory Science, College of Medicine and Health Sciences, Samara University, Samara, Ethiopia; Menzies School of Health Research, AUSTRALIA

## Abstract

**Background:**

Malaria is among the leading causes of mortality and morbidity among under five children in developing countries. Ethiopia has set targets for controlling and eliminating malaria through at-risk group interventions. However, the disease remains a serious public health concern in endemic areas like in Wollo, Northeast Ethiopia. Therefore, this study aimed to determine malaria prevalence, risk factors and parasite density among under five children in Ziquala district.

**Method:**

A facility—based cross-sectional study was conducted in Ziquala hospital, and Tsitsika, Mishra and Hamusit health centers in Ziquala district, Northeast Ethiopia, from January 2022 to April 2022. The study enrolled a total of 633 under five children using a systematic sampling technique. A capillary blood sample was collected from each child to prepared thin and thick blood smears. Smears were then stained with 10% Giemsa and examined under light microscope. A pretested structured questionnaire was used to collect on socio-demographic data, parental/caregiver knowledge, and malaria determining factors. Bivariable and multivariable logistic regression analysis was done to identify factors associated with malaria.

**Result:**

The overall prevalence of malaria among children visiting Ziquala district health institutions was 24.6% (156/633). *Plasmodium falciparum*, *P*. *vivax*, and mixed infection (both species) accounted for 57.1%, 38.5%, and 4.5% of the cases, respectively. Regarding to parasite load, moderate parasitemia was the most common, followed by low and high parasitemia with the proportion of 53.8%, 31.4% and 14.7% parasite density, respectively. Malaria infection was linked to irregular utilization of insecticide-treated bed nets (AOR = 5.042; 95% CI: 2.321–10.949), staying outside at night (AOR = 2.109; 95% CI: 1.066–4.173), and parents not receiving malaria health education in the past six months (AOR = 4.858; 95% CI: 2.371–9.956).

**Conclusion:**

Malaria was prevalent among children under the age of five enrolled in the study. The local government should focus on regular insecticide treated net utilization, reducing the risk of mosquito bites while sleeping outdoors at night and increasing public understanding of malaria prevention and control through health education would also help to minimize the burden of malaria.

## Introduction

Malaria is one of the most serious public health concerns and a life threatening parasitic disease caused by the *Plasmodium* species [1]. Malaria is the most common cause of death in children under the age of five years and pregnant women in developing countries [2–4]. Children are one of the most vulnerable groups because they are immunologically naive to malaria parasites. Malaria may cause as many as 10% of all deaths in children globally. Severe anemia, respiratory distress, and cerebral malaria are common complications in children with severe malaria [5].

Between 2000 and 2019, global malaria prevention and control activities were ramped up, and the World Health Organization African Region achieving significant success in reducing its malaria burden, particularly in Sub-Saharan Africa [6]. However, in tropical and subtropical areas of the world, malaria remains a major concern. The World Health Organization (WHO) 2021 malaria report indicates that the WHO African Region continues to bear the largest burden of malaria. In 2020, the African Region was accounted for 95% of all malaria cases (228 million); 96% of all malaria deaths (602 000); and 80% of all malaria deaths in the region were among children under the age of five. Malaria services were disrupted during the Covid-19 pandemic starting in 2020, adding to the region’s malaria burden [7].

Despite strong investments to decrease malaria incidence rates over the past years, infection rates remain significant in sub-Saharan Africa. Children under the age of five in sub-Saharan Africa, where the great majority of malaria deaths occur each year, are particularly vulnerable to the disease [7]. Every day, over 1200 children die from malaria in this region, accounting for the majority of the continent’s 631,000 child deaths [8]. With about 75% of Ethiopia’s landmass estimated to be malarious, 68% of the country’s population is at risk of malaria infection. According to Ethiopian Malaria Indicator Survey 2015, malaria is one of the top ten primary causes of morbidity. *Plasmodium falciparum* causes 60% of malaria cases, while the rest is caused by *P*. *vivax* [9].

To prevent and reduce malaria infections, several intervention activities such as the distribution of insecticide-treated nets (ITNs), indoor residual spraying (IRS), artemisinin-based combination therapy (ACT), and health information dissemination have been carried out in Ethiopia. These efforts notwithstanding, malaria remains a serious public health concern in endemic areas, particular among children under the age of five [10]. Ethiopia is implementing a malaria elimination program with the goal of eliminating the disease by 2030 [11]. To assess the progress of the program, the prevalence and determinants of malaria among vulnerable groups should be evaluated over time and in different areas.

Malaria is one of the top causes of morbidity and mortality in both adults and children under the age of five in Ziquala district in northeast Ethiopia, according to a district health office report. However, a study on the epidemiology of malaria in children under the age of five had not been conducted.Knowing the prevalence, parasite density, and determinants of malaria in children under the age of five in health facilities is critical for scaling up and designing effective intervention programs. Therefore, this study fills in an important knowledge gap by identifying potential risk factors for malaria infection among under five-year children in Ziquala District.

## Materials and methods

### Study design, area, and period

A multi-site facility-based cross-sectional study was conducted in Ziquala hospital, and Tsitsika, Mishra and Hamusit health centers in Ziquala district, Northeast Ethiopia, from January 2022 to April 2022. Ziquala district is one of the eight districts of Wag Hemra Zone, which is located at a distance of 780 km from capital city of the country, Addis Ababa. The district has an altitude of 1462 meters above sea level. Its annual average rainfall and temperature are 255 mm and 22°C, respectively [12]. The area’s major and minor transmission seasons take place between September and December and between April and May, respectively [13]. Malaria infection is primarily due to *P*. *falciparum*. Subsistence farming and livestock breading are the main occupations in the area. Water conducive to mosquito breeding is available throughout the year because of rivers such as the Tekezie and Tela rivers. In the district, there is one primary hospital, five health centers, and fifteen health posts. All health facilities provide diagnostic and treatment services to the community.

### Sample size determination and sampling technique

The sample size was calculated using a single population proportion formula, the 95% confidence limits (Zα/2 = 1:96), and 5% margin of error (d) with a maximum proportion of 50%.

Sample size = Z(α/2)^2^ P (1- P)/ d^2^, (1.96)^2^ *0.221 (1–0.221)/ (0.05)^2^ = 384.

Accounting for a 10% non-response rate and implementing design effect of two, the final sample size was 633. Study health facilities were chosen by stratifying the district’s health facilities as malaria-endemic and non-endemic based on their altitude. Then, among the resulting six health facilities in the district, four were selected for data collection by simple random sampling technique. The number of study participants to be enrolled at each health facility was determined based on the number of febrile under five children at each health facility during the same period (January 2021 to April 2021) of the previous year. Hence in this period, a total of 1240 febrile under five children were screened for malaria from Ziquala Hospital (482), and Tsitsika (376), Mishra (251) and Hamusit (131) health centers. Accordingly 246, 192, 128 and 67 febrile patients were included from Ziquala Hospital and Tsitsika, Mishra and Hamusit health centers, respectively. Finally, a systematic random sampling was applied to select study participants at each health facility.

### Study population and eligibility criteria

The source population consisted of all children under the age of five who visited the designated health facilities during the study period. The study only included children whose parents or guardians signed a written consent form. Children who had undergone antimalarial chemotherapy 42 days before to data collection were excluded because the parasite is thought to be cleared from the blood within 42 days of treatment, and re-infection can happen after that.

### Data collection

#### Socio-demographic and associated risk factors assessment

To collect data on socio-demographic and related risk factors for malaria, a standardized questionnaire was developed in English. It was then translated into Agewgna, the indigenous language. Data of socio-demographic and environmental were collected from their parents/caregivers using structured and pretested questionnaire by trained data collectors.

### Laboratory data collection

#### Blood sample collection, processing, and examination

At Ziquala hospital, and Tsitsika, Mishra and Hamusit health centers laboratories; capillary blood was drawn aseptically from the children’s fingers using a blood lancet. Thin and thick blood films were prepared and air dried. After that, the thin blood film was fixed with absolute methanol and allowed to air dry. Both thin and thick blood films were stained with 10% Giemsa stain for 10 minutes. The slides were then air dried after being washed with distilled water. Finally, laboratory technologists at the health facilities examined the stained slides. Thick smears were used to detect *Plasmodium* infections, while thin smears were employed for parasite species identification using an oil immersion objective.

To measure parasitemia, the asexual forms of the parasites, such as trophozoites and schizonts, were manually counted until 200 WBCs from thick smear using a tally counter. The number of parasites per microliter of blood was calculated using the formula below, with the patient’s total leukocyte count assumed to be 8000/μl of blood. Parasite load is equal to the parasites counted per 200WBC X 8000WBC/μl blood. The parasitemia was then classified as low (<1000 parasites per microliter of blood), intermediate (1000–4999 parasites per microliter of blood) and high (>5000 parasites per microliter of blood) [14].

### Data quality assurance

Data collectors were trained for two days by the investigators. The Giemsa stain was tested on known positive and negative malaria slides. During the microscopic examination, a slide was regarded as negative after 200 fields had been examined without finding of *Plasmodium* parasite by two laboratory technologists at each site. A colour atlas was used during microscopic examination. To assure quality of the microscopic examinations, all positive and 10% of the negative slides were re-examined by a third reader to remove discrepant result.

### Data processing and analysis

Data were entered and analysed using SPSS version 22 after data collection. The prevalence rate and determinant factors were estimated using descriptive statistics. Bivariable and multivariable logistic regression models were used to find correlations with malaria infection. The multivariable logistic regression analysis included variables with a p-value of less than or equal to 0.25 in the bivariable regression. A p-value <0.05 was considered to determine statistical significance. Finally, the strength of the association between factors was determined using adjusted odds ratios (AOR) with a 95% confidence interval (CI).

## Results

### Socio-demographic characteristics of study participants

A total of 633 febrile children under the age of five years old took part in the study, with a 100% response rate. Of the total participants more than half (53.9%) of individuals were males and 46.1% of them were females. Regarding to their age group [15], the highest number (33.2%) of children participated were in the age between 12 and 23 months. The majority of the children were come from rural areas (76.0%), while the rest from urban areas (Table 1).

**Table 1:** Socio-demographic characteristics of children under five visiting health institutions in Ziquala district, Northeast Ethiopia, 2022.

Variable	Categories	Number (%)
1. Sex	1. Male	341(53.9)
	2. Female	292 (46.1)
2. Age in months	1. <12	152 (24.0)
	2. 12–23	210 (33.2)
	3. 24–35	109 (17.2)
	4. 36–47	94 (14.8)
	5. 48–59	68 (10.7)
3. Residence	1. Rural	481 (76.0)
	2. Urban	152 (24.0)

### Prevalence of malaria and its density

The overall prevalence of malaria among under-five children in Ziquala district was 24.6% (95% CI = 21.1–28.1). Of these, 60.3% were males and 39.7% were females. Malaria prevalence in sex was not statistically significant difference (P = 0.065). Likewise, the higher number of malaria cases was detected a mong the age group of 12–23 and less than 12 months with the prevalence of 45.5% and 24.4%, respectively. *Plasmodium* parasite prevalence was higher in younger children, with a statistically significant difference (P = 0.001). The malaria parasite was shown to be more prevalent in children living in rural areas as compared to children in urban areas. Home village location was not associated with malaria infection (p = 0.455). In this study, the prevalence of *P*. *falciparum* and *P*. *vivax* was 57.1% and 38.5%, respectively, while that of mixed infection was 4.5% (Table 2).

**Table 2:** Socio-demographic variables and malaria infection among children under five years in Ziquala district, Northeast Ethiopia, 2022.

Variable	Categories	N	Positive n (%)	*P*. *falciparum* n (%)	*P*. *vivax* n (%)	Mixed n (%)	Negative n (%)	P-value
Sex	Male	341	94 (27.6)	56 (59.6)	34(36.2)	4(4.3)	247(72.4)	0.065
	Female	292	62 (21.2)	33 (53.2)	26(42.0)	3(4.8)	230(78.8)	
Age in months	<12	152	38 (25.0)	24 (63.2)	14(36.8)	0(0)	114(75.0)	0.001
	12–23	210	71 (33.8)	39 (54.9)	31(43.7)	1(1.4)	139(66.2)	
	24–35	109	24 (22.0)	15 (62.5)	6(25.0)	3(12.5)	85 (78.0)	
	36–47	94	15(16.0)	7(46.7)	6(40.0)	2(13.3)	79 (84.)	
	48–59	68	8 (11.8)	4 (50.0)	3 (37.5)	1(12.5)	60(88.2)	
Residence	Rural	481	122 (25.4)	72 (59.0)	45(36.9)	5(4.1)	359(74.6)	0.455
	Urban	152	34 (22.4)	17 (50.0)	15(44.1)	2(5.9)	118(77.6)	
Total		633	156 (24.6)	89 (57.1)	60(38.5)	7(4.5)	477(75.6)	

Regarding to parasite density, moderate parasitemia was the most common, followed by low and high parasitemia, which accounted for 53.8%, 31.4%, and 14.7% individuals respectively. Males were found to have a higher prevalence of moderate malaria parasitemia than females, accounting for 57.1% of the total, however there was no statistical difference in parasite density (p = 0.683). Likewise, moderate parasitemia had the highest parasite density in the 12–23 age group, and there was statistical significance in parasitemia across age groups (p = 0.001). Moreover, there was a statistically differences (p = 0.001) between rural and urban residents, with 70.2% of rural residents having moderate parasitemia (Table 3).

**Table 3:** Distribution of malaria parasite density by age, sex, and residence among children visiting health institutions in the Ziquala district, 2022.

Variable	Categories	Parasite density				
		Low (%)	Moderate (%)	High (%)	Total (%)	P-value
Sex	Male	31 (34.0)	48 (51.1)	15 (16.0)	94 (60.3)	0.683
	Female	18 (29.0)	36 (58.1)	8 (12.9)	62 (39.7)	
	Total	49 (31.4)	84 (53.8)	23 (14.7)	156 (100)	
Age in months	<12	24 (63.2)	14 (36.8)	0 (0.0)	38 (24.4)	0.001
	12–23	14 (19.7)	45 (63.4)	12 (16.9)	71 (45.5)	
	24–35	11 (45.8)	10 (41.7)	3 (12.5)	24 (15.4)	
	36–47	0 (0.0)	11 (73.3)	4 (26.7)	15 (9.6)	
	48–59	0 (0.0)	4 (50.0)	4 (50.0)	8 (5.1)	
	Total	49 (31.4)	84 (53.8)	23 (14.7)	156 (100)	
Residence	Rural	47 (38.5)	59 (48.4)	16 (13.1)	122 (78.2)	0.001
	Urban	2 (5.9)	25 (73.5)	7 (20.6)	34 (21.8)	
	Total	49 (31.4)	84 (53.8)	23 (14.7)	156 (100)	

The highest numbers of children who participated were from Ziquala hospital (38.9%), while the lowest were from Hamusit health center (10.6%). There was also a considerable variation in the prevalence rate between the health facilities, ranging from 19.4% at Hamusit health center to 28.0% at Ziquala hospital (Table 4).

**Table 4:** Malaria distribution by different health institutions in Ziquala district, Northeast Ethiopia from January to April 2022.

Health institution	Blood films Examined Number (%)	Confirmed cases Number (%)	Plasmodium species		
			*P*. *falciparum* Number (%)	*P*. *vivax* Number (%)	Mixed Number (%)
Ziquala hospital	246 (38.9)	69 (28.0)	42 (60.9)	24 (34.8)	3 (4.3)
Tsitsika health center	192 (30.3)	47 (24.5)	27 (57.4)	17 (36.2)	3 (6.4)
Mishra health center	128 (20.2)	27 (21.1)	15 (55.6)	11 (40.7)	1 (3.7)
Hamusit health center	67 (10.6)	13 (19.4)	5 (38.5)	8 (61.5)	0 (0.0)
Total	633(100)	156 (24.6)	89 (57.1)	60 (38.5)	7 (4.5)

### Prevalence of malaria and knowledge of parents/caregivers

In this study, 72.0% of the parents/caregivers had no ability to read or write, whereas 28.0% had attained various levels of formal education. Of the total parents/caregivers, 91.9% believed that malaria is a curable disease and 72.0% had knowledge of malaria transmissibility between individuals. The majority of the respondents (78.0%) were aware that mosquitoes are a vector for malaria transmission, whereas the remaining (22.0%) had misconceptions about the mode of transmission. Despite the high prevalence of malaria, 70.3% of participants had knowledge about the breeding site of mosquitoes. Meanwhile, 94.0% of the respondents knew that malaria is a preventable disease. When asked about malaria prevention measures, the majority of the respondents 73.0% mentioned that insecticide treated nets (ITNs) as a malaria preventive method while only 2.5% mentioned that malaria can be prevented through indoor residual spraying (IRS). On the other hand, children under five and pregnant women were accurately identified as the most susceptible segments of the population by 32.5% and 19.6% of the respondents, respectively. Furthermore, a majority (84.0%) of the children sought treatment at least within 24 hours after the onset of fever. Early diagnosis and treatment, within 24 hours after the onset of symptoms, were recognized as critical components of malaria control (Table 5).

**Table 5:** Socio-demographic characteristics and knowledge of parents/caregivers with the distribution of malaria among children under five years, in Ziquala district, Northeast Ethiopia.

Knowledge	Variables	Total n (%)	Positive n (%)	Negative n (%)
Educational status	Illiterate	456 (72.0)	123 (27.0)	333 (73.0)
	Primary education	142 (22.4)	27 (19.0)	115 (81.0)
	Secondary education	23 (3.6)	4 (17.4)	19 (82.6)
	Higher education	12 (1.9)	2 (16.7)	10 (83.3)
Malaria is curable	Yes	582 (91.9)	142 (24.4)	440 (75.6)
	No	51 (8.1)	14 (27.5)	37 (72.5)
Malaria is transmittable	Yes	456 (72.0)	108 (23.7)	348 (76.3)
	No	64 (10.1)	16 (25.0)	48 (75.0)
	I do not know	113 (17.9)	32 (28.3)	81 (71.7)
Method of transmission	Mosquito bite	494 (78.0)	118 (23.9)	376 (76.1)
	Others	139 (22.0)	38 (27.3)	101 (72.7)
Breeding site of mosquitoes	Stagnant water	445 (70.3)	111 (24.9)	334 (75.1)
	Running water	118 (18.6)	29 (24.6)	89 (75.4)
	Soil	19 (3.0)	4 (21.1)	15 (78.9)
	Do not know	51 (8.1)	12 (23.5)	39 (76.5)
Malaria can be prevented	Yes	595 (94.0)	144 (24.2)	451 (75.8)
	No	38 (6.0)	12 (31.6)	26 (68.4)
Method of malaria prevention	ITNs	462 (73.0)	102 (22.1)	360 (77.9)
	IRS	16 (2.5)	4 (25.0)	12 (75.0)
	Drug	45 (7.1)	9 (20.0)	36 (80.0)
	Environmental mgt	102 (16.1)	39 (38.2)	63 (61.)
	Don’t know	8 (1.3)	2 (25.0)	6 (75.0)
Group of people more affected by malaria	under-five children	206 (32.5)	32 (15.5)	174 (84.5)
	Pregnant women	124 (19.6)	25 (20.2)	99 (79.8)
	Adults	76 (12.0)	28 (36.8)	48 (63.2)
	All are equally affected	227 (35.9)	71 (31.3)	156 (68.7)
Treatment seeking within 24 hours	Good	532 (84.0)	125 (23.5)	407 (76.5)
	Poor	101 (16.0)	31 (30.7)	70 (69.3)

### Factors associated with malaria infection

In this study, 94.0% of the respondents possessed an ITNs, and 96.1% had received IRS service in the previous 12 months. In terms of ITNs utilization, 58.0% of under-five children slept in an ITNs on a regular basis during the previous two weeks. In bi-variable logistic regression, the following variables were chosen and entered into the forward stepwise multivariable logistic regression model: stagnant water near the house, under five children sleeping under ITNs regularly for the last two weeks, number of under five children per household, staying outside at night, and health education on malaria in the previous six months.

In the final adjusted model, children under the age of five who did not sleep under an ITNs regularly for the last two weeks were more likely to be infected with malaria (adjusted OR [AOR] = 5.042; 95% CI: 2.321–10.949) than those who did. Children who stayed outside at night had a 2.1-fold increased chance of contracting malaria than children who did not (AOR = 2.109, 95% CI: 1.066–4.173). In addition, children whose mother/caregiver had never received malaria health education in the previous 6 months also had a greater risk of infection (AOR = 4.858, 95%; CI = 2.371–9.956) than those whose mother/caregiver had received malaria health education (Table 6).

**Table 6:** Bivariable and multivariable logistic regression for determinants factors of malaria infection among children under five years, in Ziquala district, Northeast Ethiopia, 2022.

Variables	Category	Malaria		COR (95%CI)	P-value	AOR (95%CI)	P-value
		Yes; N (%)	No; N (%)				
Place of residence	Rural	122 (25.4)	359 (74.6)	1.179 (0.765–1.189)	0.455		
	Urban	34 (22.4)	118 (77.6)	1			
History of malaria	Yes	46 (27.5)	121 (72.5)	1.230 (0.824–1.838)	0.311		
	No	110 (23.6)	356 (76.4)	1			
Stagnant water near to the house.	Yes	58 (36.3)	102 (63.7)	2.179 (1.471–3.219)	0.001	1.764 (0.798–3.428)	0.062
	No	98 (20.7)	375 (79.3)	1			
Distance from Health center	≤1 hr	62 (25.2)	184 (74.8)	1			
	>1hr	94 (24.3)	293 (75.7)	0.952 (0.658–1.378)	0.795		
Presence of ITN	Yes	144 (24.2)	451(75.8)	1			
	No	12 (31.6)	26 (68.4)	1.446 (0.711–2.938)	0.314		
Under five children sleeping under ITN regularly for the last 2 weeks	Yes	52 (14.2)	315 (85.8)	1			
	No	104 (39.1)	162 (60.9)	3.889 (2.651–5.704)	0.001	5.042 (2.321–10.949)	0.001
Number of U5 children/HH	1	91 (20.6)	351 (79.4)	1			
	>2	65 (34.0)	126 (66.0)	1.990 (1.364–2.903)	0.001	0.198 (0.091–0.431)	0.431
IRS service in the last 12 months	Yes	152 (25.2)	456 (74.8)	1			
	No	4 (15.4)	21(84.6)	0.57 (0.193–1.691)	0.312		
Outdoor stay at night	Yes	32 (42.1)	44 (57.9)	2.540 (1.545–4.175)	0.001	2.109(1.066–4.173)	0.032
	No	124 (22.3)	433 (77.7)	1			
Presence of hole on the wall of house	Yes	47 (29.7)	111(70.3)	1.42(0.951–2.127)	0.087		
	No	109 (22.9)	366 (77.1)	1			
Health education on malaria in the last 6 months	Yes	103 (19.8)	417 (80.2)	1			
	No	53 (46.9)	60 (53.1)	3.576 (2.331–5.485)	0.001	4.858 (2.371–9.956)	0.001

## Discussion

The present study showed that malaria is a public health concern among children under five years old in the study area. The overall prevalence of malaria was 24.6% (95% CI = 21.1–28.1) among children visiting health institutions in Ziquala district. This result was comparable to a study conducted in Arsi Negele, Ethiopia (22.8%) [16], Arba Minch Zuria District, South Ethiopia (22.1%) [17] and Nigeria (22.6%) [18]. In contrast, the prevalence determined in this study showed a lower prevalence of malaria than a study conducted in Mali (35.4%) [19], Ghana (43.0%) [20] and Cameron (28.8%) [21]. The low prevalence of malaria parasites in this study could be due to seasonal variations, as the current study was conducted in a minor malaria transmission season. This difference might also be due to the difference in geographical variation, and malaria control and prevention programs implemented in the study areas.

However, the prevalence of malaria in this study was higher than the prevalence determined by studies conducted in Wogera district, Ethiopia (8.7%) [15], Sherkole refugee camp, Ethiopia (3.9%) [22] and the national magnitude of malaria among under-five children (0.6%) [13], Sudan (18.0%) [23] and Uganda (19.5%) [24]. This could be due to the community’s and responsible bodies’ attention being diverted to the Covid-19 pandemic, which has resulted in less attention being paid to malaria prevention and control activities. Such an assertion is in accordance with the WHO global malaria report 2021 [7], which states that delays to malaria services during the Covid-19 pandemic had an additional influence on the malaria burden. In East Africa, the growth of antimalarial drug resistance is also a major problem [25, 26]. This high prevalence of malaria in the study area proves that the efforts that have been made by the district, as well as the regional health office and other concerned bodies to control malaria in the study area, were not sufficient.

*Plasmodium falciparum* and *P*. *vivax* were the two species identified in the blood of the children, accounting for 57.1% and 38.5% of infections, respectively. This result is in line with the national *Plasmodium* species distribution [27] and other previous studies done in Ataye [28], Kombolcha [29] and Dembecha [30], Ethiopia. The higher dominance of *P*. *falciparum* over *P*. *vivax* can be explained by several factors. These include *P*. *falciparum*’s high proliferation in the host cell, the parasite’s capacity to infect all ages of red blood cells, the parasite’s resistance to first line treatment [31] and the study area’s has lowland climatic conditions, where *P*. *falciparum* is a widespread species in the lowlands.

In this study similar to a study done in Wogera district, Ethiopia [15], the number of children who had febrile illness and *Plasmodium* infection decreases with increasing age of a child. This is might be due to these children live in stable malaria transmission endemic areas and can develop age-related protective immunity as the result of continuous exposure to infective mosquito bites [32]. The majority of infected children had a moderate parasite density, followed by low and high parasite density, which accounted for 71%, 16% and 12.9% of malaria positive children respectively. This is consistent with an earlier study done in South Gondar, Ethiopia [33]. However, a high proportion of high parasite density was found in East Central Tanzania [34], while a high proportion of low parasite density was found in Sanja Town [35]. The immunological conditions, age category, and dietary status of the study participants could all have an impact on parasite density. By decreasing parasite load, acquired or adaptive immunity protects against clinical disease, morbidity associated with parasite density, and new infection [36].

Regarding to the health institutions, Ziquala hospital has the highest rate of malaria infection, followed by Tsitsika and Mishra health centers. *Plasmodium falciparum* was the predominant species found in all health facilities except Hamusit health center. This variability in the prevalence of malaria among different locations is in line with studies undertaken in Wolkite, Ethiopia [37], lowlands of southern Ethiopia [38] and Cameron [39]. This could be due to differences in malaria prevention and control interventions from one area to another. These variations could also be due to altitude differences and climate diversity which affect the reproduction of the Anopheles vector.

About 78.0% of the parents/caregivers had knowledge that mosquitoes are a vector for the transmission of malaria. This result was relatively lower than other studies done in Damot Gale, Wolayta Zone (85.0%) and Gondar (94.5%) Ethiopia [40, 41]. However, knowledge of causes of malaria in this site was better than a study done in Gilgel Gibe, Ethiopia (70.1%) [42]. This difference might be due to endemicity of malaria and related efforts to control it among vulnerable population in the area. Among the study participants, 70.3% knew that stagnant water is a suitable breeding site for mosquitoes. Several studies in Ethiopia [15, 22, 33] have showed that malaria vector density and living close to a water body like a river or streams could be an important factors influencing malaria transmission.

When asked about malaria prevention measures, 73.0% of responders identified ITNs, whereas only 2.5% chose IRS. The knowledge of using ITNs (73.0%) and IRS (2.5%) for malaria prevention was lower than a study done in Damot Gale, Ethiopia [40]. According to WHO malaria control and elimination strategies, access to all interventions enhance reduction in malaria, including enhanced case management, scale-up of ITNs, IRS, early diagnosis and treatment, and environmental management [43]. The majority of the children (84.0%) sought treatment at least within 24 hours after the onset fever. Early detection and treatment, within 24 hours of the onset of symptoms are an essential element of malaria control. This finding was higher than the same figure in Damot Gale (71.7%) and Mandura (38.7%) Districts, Ethiopia [40, 44]. This increased health-seeking behaviour within 24 hours could be due to the presence of good awareness towards prompt access to antimalarial drugs.

This study revealed that those children who did not reguraly use ITNs were 5.04 times more likely than those who did to be infected with the *Plasmodium* parasite It was similar with prior studies conducted in South Gondar and Arbaminch Zuria District, Ethiopia [17, 33]. Insecticide-treated nets are an effective vector control method for preventing malaria transmission. When they use ITNs consistently, the risk of getting a mosquito bite might be decreased [45]. Therefore, regular ITNs usage must be enforced by local authorities in malaria endemic areas.

Children who stayed outdoors at night were more likely to be exposed from malaria infection compared to those that did not. Staying outside during the night showed a statistically significant association with malaria. This finding was in line with the previous studies done in Armachiho, in Dembia district and Sherkole refugee camp in Benishangul-Gumuz, Ethiopia [22, 46, 47]. This could be explained by exophagic-exophilic mosquito biting behavior [48]. Children whose mother/caregiver had never received malaria health education in the previous 6 months had a greater risk of infection than those whose mother/caregiver had received. This finding was also supported by the previous studies done in Uganda and Ghana [20, 49].

## Conclusion and recommendations

The prevalence of malaria in under-five children attending at selected health facilities of Ziquala district was high. The highest prevalence of malaria was found in those aged between 12 and 23 months old, and a higher proportion of moderate parasitemia was also observed in this study. Irregular use of bed net, staying outside at night and parents’ not receiving malaria health education were the main correlates of malaria. The local government and other concerned bodies should give focus on regular ITNs utilization, infection associated with staying outdoors at night, environmental management and changing attitudes towards malaria prevention and control through health education to minimize the burden of malaria.

## Supporting information

S1 Data (SAV)
